# Pretreatment with intrathecal amitriptyline potentiates anti-hyperalgesic effects of post-injury intra-peritoneal amitriptyline following spinal nerve ligation

**DOI:** 10.1186/1471-2377-12-44

**Published:** 2012-06-21

**Authors:** Kuang-I Cheng, Hung-Chen Wang, Lin-Li Chang, Fu-Yen Wang, Chung-Sheng Lai, Chao-Wen Chou, Hung-Pei Tsai, Aij-Lie Kwan

**Affiliations:** 1 Graduate Institute of Medicine, College of Medicine, Kaohsiung Medical University, Kaohsiung, Taiwan, R.O.C; 2 Department of Anesthesiology, Kaohsiung Medical University Hospital, Kaohsiung, Taiwan, R.O.C; 3 Department of Anesthesiology, Faculty of Medicine, College of Medicine, Kaohsiung Medical University, Kaohsiung, Taiwan, R.O.C; 4 Department of Neurosurgery, Chang Gung Memorial Hospital-Kaohsiung Medical Center, Chang Gung, University College of Medicine, Kaohsiung, Taiwan, R.O.C; 5 Department of Microbiology, Faculty of Medicine, College of Medicine, Kaohsiung Medical University, Kaohsiung, Taiwan, R.O.C; 6 Department of Surgery, Faculty of Medicine, College of Medicine, Kaohsiung Medical University, Kaohsiung, Taiwan, R.O.C; 7 Division of Plastic and Reconstructive Surgery, Department of Surgery, Kaohsiung Medical University Hospital, Kaohsiung, Taiwan, R.O.C; 8 Department of Neurosurgery, Kaohsiung Medical University Hospital, Kaohsiung, Taiwan, R.O.C

**Keywords:** Intrathecal amitriptyline, Allodynia, Sodium channels, Microglia, Astrocyte

## Abstract

**Background:**

Amitriptyline, a tricyclic antidepressant and potent use-dependent blocker of sodium channels, has been shown to attenuate acute and chronic pain in several preclinical modes. The purpose of this study was to investigate whether intrathecal pretreatment with amitriptyline combined with post-injury intra-peritoneal amitriptyline is more effective than post-injury treatment alone on L5 spinal nerve ligation (SNL)-induced neuropathic pain.

**Methods:**

96 adult male Sprague–Dawley rats were allocated into 4 groups: group **S**, Sham; group **L**, L5 spinal nerve **L**igation with vehicle treatment; group **A**, SNL and post-injury intra-peritoneal (**A**bdominal) amitriptyline twice daily × 3 days; group **P**, intrathecal **P**retreatment with amitriptyline, SNL and intra-peritoneal amitriptyline twice daily × 3 days. Responses to thermal and mechanical stimuli, as well as sodium channel expression in injured dorsal root ganglion (DRG) and activated glial cells in spinal dorsal horn (SDH) were measured pre-operatively and on post-operative day (POD) 4, 7, 14, 21 and 28.

**Results:**

SNL-evoked hyper-sensitivity responses to thermal and mechanical stimuli, up-regulated Nav1.3 and down-regulated Nav1.8 expression in DRG, and activated microglia and astrocytes in SDH. In group A, intra-peritoneal amitriptyline alone alleviated thermal hypersensitivity on POD7, reversed Nav1.8 and reduced activated microglia on POD14. In group P, intrathecal pretreatment with amitriptyline not only potentiated the effect of intra-peritoneal amitriptyline on thermal hypersensitivity and Nav1.8, but attenuated mechanical hypersensitivity on POD7 and reduced up-regulated Nav1.3 on POD14. Furthermore, this treatment regimen reduced astrocyte activation on POD14.

**Conclusions:**

Concomitant intrathecal pretreatment and post-injury intra-peritoneal amitriptyline was more effective than post-injury treatment alone on attenuation of behavioral hypersensitivity, decrease of activated microglia and astrocytes and dysregulated Nav1.3 and 1.8.

## Background

Peripheral neuropathic pain caused by nerve injury is a common event after trauma, amputation or surgery [1-4]. To relieve neuropathic pain after surgery, medicines are generally administered through systemic or regional routes peri-operatively [1,4]. In preclinical models, voltage-gated sodium channel (Nav) blockers administered at the site of injury and on injured dorsal root ganglion (DRG), either before or immediately after nerve damage, inhibit upward transmission of noxious stimuli and play a major role in attenuating acute and chronic neuropathic pain [5-8].

Injured DRG neurons are likely to have a more hyperpolarized threshold for overshooting action potential with the presence of Nav1.3 in place of Nav1.8 after peripheral nerve axotomy. The hyperpolarized threshold brings the neurons requiring less depolarization for activation and easy firing under the actions of Nav1.3 and Nav1.7 [9]. Amitriptyline, a tricyclic antidepressant, acts as a potent, use-dependent blocker of sodium channels at therapeutic doses [10]. Amitriptyline has also been demonstrated to attenuate acute and chronic pain after surgery [11,12]. It inhibits ectopic and bursting discharges from injured nerves and modulates activation and inactivation kinetics of sodium channels in sensory neurons [13]. In addition, systemic or intraspinal administration of amitriptyline has been demonstrated to produce an anti-hyperalgesic effects on same sensory endpoints [12,14,15]. However, the mechanism by which amitriptyline exerts its effect on the prevention of mechanical allodynia remains controversial. Peripheral administration of sodium channel blockers can alleviate mechanical allodynia [16,17] and peripheral administration of amitriptyline produces anti-allodynic effects [18]. In addition, intrathecal amitriptyline (0.21 mg in 90 μl) produces a complete motor and sensory blockade with a potent and long duration [19]. Hence, amitriptyline possesses anti-allodynic effect through its potent activity as a sodium channel blocker.

Recent reports have demonstrated that neuropathic pain is associated with dysregulation of Nav expression, namely up-regulation of Nav1.3 and down-regulation of Nav1.8 in injured DRG [20,21] as well as over-activation of microglia in the spinal dorsal horn (SDH) [22]. In view of the above reports, we hypothesized that concomitant intrathecal pretreatment with post-injury intra-peritoneal amitriptyline could attenuate nerve damage-induced hyperalgesia and allodynia. In the present study, both thermal and mechanical stimulations were used to investigate the effect of a concomitant administration of intrathecal and intra-peritoneal amitriptyline on spinal nerve ligation-induced neuropathic pain. We also evaluated the effect of amitriptyline on sodium channel expression in injured DRG, and activation of glial cells in SDH. This study found that intrathecal pretreatment in combination with post-injury intra-peritoneal amitriptyline was more effective than post-injury treatment alone on attenuation of thermal and mechanical hypersensitivity as well as reduction of microglia and astrocyte activation in SDH, and reduction of dysregulated Nav1.3 and Nav1.8 in DRG.

## Methods

### Experimental animals and groups

**Figure 1:**
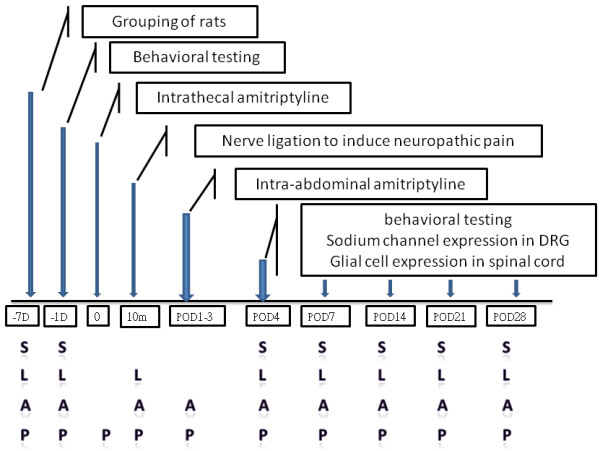
**Flow chart of the study.** DRG: dorsal root ganglion; D: day; m: minute; POD: postoperative day; S: **S**ham; L: **L**igation followed by vehicle treatment; A: ligation followed by treatment with amitriptyline intra-peritoneally post-injury; P: **P**retreatment intrathecal amitriptyline, ligation and post-injury intra-peritoneal amitriptyline.

Ninety-six adult male Sprague–Dawley rats weighing 280–300 g were used. Ethical approval for this study (Approval No. 95105) was provided by the Institutional Animal Care and Use committee, Kaohsiung, Taiwan. The experimental rats were allocated into four groups (n = 24/group) (Figure 1): (1) sham group (group S), removal of left sixth lumbar transverse process; (2) ligation group (group L), surgery with left L5 spinal nerve ligation (SNL) followed by treatment with vehicle; (3) intra-peritoneal (**A**bdominal) group (group A), left L5 SNL followed by treatment with amitriptyline intra-peritoneally at 12.5 mg/kg twice daily for 3 days after nerve injury; (4) Pretreatment group (group P), pretreatment with 90 μl of 7.5 mM (0.21 mg in 90 μl) amitriptyline hydrochloride (Sigma) intrathecally followed by left L5 SNL, and then treatment with amitriptyline intra-peritoneally at 12.5 mg/kg twice daily for 3 days after nerve injury. Behavioral testing to noxious thermal stimuli and to mechanical stimuli applied to the hind paws was performed in all rats on day −1 and on post-operative days 4 and 7 (POD4 and POD7) and then weekly thereafter for the next 3 weeks (POD14, POD21_,_ and POD28)_._

### Intrathecal injection of amitriptyline

Following an optimal flexion of the lumbar spine in a prone position, 100 μl of 1% lidocaine was injected subcutaneously into the lumbar 4–5 (L4-5) intervertebral space. After local infiltration, 90 μl of 7.5 mM amitriptyline was administered by intrathecal injection through the L4-5 intervertebral space using a 100 μl syringe with a 30-gauge needle (Hamilton, Reno, NV, USA). Amitriptyline injection was considered successful if dragging of hind limbs during movement was observed [19].

### Spinal nerve ligation

All surgical procedures were performed under isoflurane/O_2_ anesthesia. Each rat was placed in a prone position, and the left paraspinal muscles were separated from the spinous processes at the L4-S2 level. The left L6 transverse process was removed with a small Rongeur to expose the L4 and L5 peripheral spinal nerves, and the L5 spinal nerve was isolated and tightly ligated with 6–0 Dexon. The wound was then sutured with 3–0 silk thread. In group S, all surgical procedures were identical except for ligation of the left L5 spinal nerve. Based on time course responses reported by Chen et al. [19], SNL surgery was performed 10 minutes after successful intrathecal amitriptyline administration in group P.

### Intra-peritoneal (abdominal) administration of amitriptyline

In this study, rats of group A and group P were administered intra-peritoneal amitriptyline hydrochloride in saline (50 mg/ml, Sigma, Taufkirchen, Germany) at 12.5 mg/kg twice daily for 3 days. In group L, rats were administered isotonic saline intra-peritoneally twice daily for 3 days.

### Behavioral responses

#### Latency of foot withdrawal in response to noxious heat stimuli

The latency of foot withdrawal from noxious heat stimuli was measured using the method described previously [23]. Briefly, an infrared light beam emitted from a moveable light box was projected through a hole (2 × 5 mm) to heat the glass plate under one hind paw (Ugo Basile Model 7370, Italy). Abrupt lifting, withdrawal, licking of the hindpaw or guarding posture was considered a positive response. A photocell was used to turn off the light beam automatically when the rat lifted the paw. The time from application of light beam to lifting of the hind paw was recorded and was defined as foot withdrawal latency. Measurements were performed at five-minute intervals and repeated 5 times on each hind paw, alternating between the two paws.

### Force of foot withdrawal response to mechanical stimuli

Rats were first acclimated to the environment for testing mechanical stimuli, which was a metal mesh floor covered by a transparent plastic dome (8 × 8 × 18 cm). A Dynamic Plantar Aesthesiometer (UgoBasil, Italy) filament underneath the metal mesh was applied perpendicular to the outer mid-plantar surface of the paw. An automated test machine was used to apply mechanical stimuli with a 2 mm diameter metal rod in increments of 2.5 g/s (to a maximum of 50 g) to either hind paw until an abrupt foot withdrawal was elicited. When rapid withdrawal of the paw was observed, duration and force intensity were recorded with approximately 0.1 g sensitivity. For each hind paw, measurements were repeated 5 times at intervals of approximately 3 minutes. The paw withdrawal force was determined by averaging measurements for each hind paw.

### Determination of protein expression

On POD4_,_ POD7, POD14_,_ POD21 and POD28_,_ the left L5 DRGs were removed from rats in all 4 groups (n = 4-5/group for each time point). The tissues were homogenized in RIPA buffer (50 mM Tris, pH 7.4, 150 mM NaCl, 1 mM EDTA, 0.1% SDS, 1% NP-40, 0.5% sodium deoxycholate) containing complete protease inhibitor mixture (Roche Diagnostics GmbH, Mannheim Germany). Fifty micrograms of total protein was loaded onto 8% sodium dodecyl sulfate-polyacrylamide gels (SDS-PAGE) and were transferred to polyvinylidene fluoride membranes (PVDF, Millipore, Bedford, MA). The expressions of Nav1.3, Nav1.7 and Nav1.8 protein were detected using rabbit primary antibodies (Alomone Labs, Jerusalem, Israel) followed by reaction with horseradish peroxidase-conjugated mouse anti-rabbit antibody (Santa Cruz, Biotechnology, Santa Cruz, CA). The intensity of each band was visualized by ECL Western blotting detection reagents (Amersham Biosciences, Tokyo, Japan). Protein expressions were normalized using β-actin as internal control, and quantification of Nav level at each time point for each group were normalized against the Nav levels of the sham group.

### Tissue preparation and immunofluorescence of microglia and astrocytes in the spinal cord

Activation of spinal microglia and astrocytes in L5 SDH were evaluated on POD4_,_ POD7, POD14_,_ POD21, and POD28. Rats from each group (n = 4-5/group for each time point) were anesthetized with thiopentone (60 mg/kg) and perfused with 0.9% saline followed by 4% paraformaldehyde in 0.1 M phosphate buffer (pH 7.4). The L5 spinal cord tissues were dissected out, fixed in 4% paraformaldehyde then saturated in 10 to 30% sucrose in 0.02 M phosphate-buffered saline (pH 7.4). The tissues were embedded in optimum cutting temperature (O.C.T.) compound, and 16-μm thick sections were cut for immunostaining. To visualize the proliferation of microglia in the spinal cord, mouse monoclonal anti-OX-42 antibody (mouse anti-rat-cd 11, 1:200, Serotec, Oxford, UK) and goat anti-mouse IgG Alexa Fluor 488 (Invitrogen, UK) secondary antibody were used. In addition, the primary anti-glia fibrillary acidic protein antibody (1:200; Millipore, Temecula, CA) and Cy3- conjugated goat anti-rabbit secondary antibody were used to detect the activation of astrocytes. Quantification of immunofluorescence staining in the spinal cord was performed by means of a computerized imaging system to analyze positive staining for microglia or astrocytes on the ipsilateral side of the dorsal horn of the spinal cord. Six sections were evaluated in each rat, and average density of microglia and astrocytes in each group was obtained.

### Histological staining

Spinal cord specimens were collected, fixed in 10% formalin in neutral buffer for several hours, embedded in paraffin, sliced to a thickness of 3–4 μm, stained with hematoxylin and eosin (H&E), and viewed under a microscope.

### Statistical analysis

Group differences in behavioral and Western blot data were compared by ANOVA followed by Scheffe test of multiple *post hoc* analyses. Microglia and astrocytes data were analyzed by Mann–Whitney *U* test. These statistical tests were performed using SPSS 18.0 (SPSS Inc., USA).

## Results

### Surgery and histo-pathologic assessment

After surgery, no rats exhibited ventroflexion or dragging of the hind paw during forward movement, and there were no signs of autotomy. In sham rats, ipsi-(left) and contra-lateral (right) behavioral responses were similar, and there was no significant difference in withdrawal latency between left and right hind paws. In addition, intrathecal treatment with amitriptyline did not show obvious or extended infiltration of inflammatory cells in the spinal cord in group P (results not shown).

### Amitriptyline blunted hypersensitive responses to thermal and mechanical stimuli after SNL

**Figure 2:**
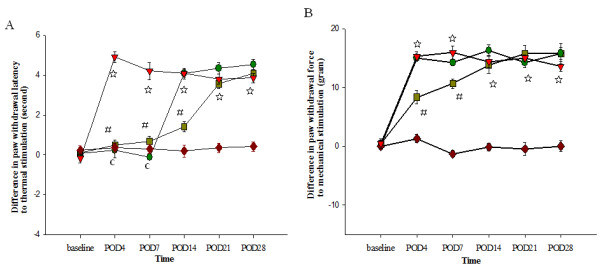
**Differences in withdrawal latency between left and right hind paws after thermal (A) and mechanical (B) stimulation.** There are no significant differences in withdrawal latencies between hindpaws in the sham group (S). Spinal nerve ligation (group L) produced a significant difference in withdrawal latencies between hindpaws. Intra-peritoneal amitriptyline post-injury (group A) alleviated thermal hypersensitivity for one week. Pretreatment intrathecal amitriptyline in combination with post-injury intra-peritoneal amitriptyline (group P) alleviated thermal hypersensitivity for 2 weeks and attenuated mechanical hypersensitivity for 1 week. POD: postoperative day, (*red accent diamond*: group S, *red down-pointing triangle*: group L, *green circle*: group A, *olive green square*: group P, n = 24/group, 4-5/each time point). L vs. S, ☆: *P* < 0.001; A vs. L, c: *P* < 0.001; P vs. L,#: *P* < 0.001. Group differences were compared by ANOVA followed by Scheffe test of multiple *post hoc* analyses. Error bars represent SE.

Spinal nerve ligation induced thermal and mechanical hypersensitivity as indicated by a significant difference in withdrawal latency and withdrawal force between left and right hind paws to both thermal and mechanical stimuli, respectively (Figure 2, group S vs. group L, *p* < 0.001). Intra-peritoneal amitriptyline alone blunted the hypersensitive responses to thermal stimuli for one week, but had no effect on mechanical responses (Figure 2, group A vs. group L). Pretreatment with intrathecal amitriptyline in addition to the intra-peritoneal regimen prolonged the effect of attenuating thermal hypersensitivity for 2 weeks (Figure 2A, *p* < 0.001) and reduced mechanical hypersensitivity for 1 week (Figure 2B, *p* < 0.001). However, regardless of the treatment with amitriptyline, the thermal and mechanical hypersensitivity responses in group A and group P have comparable results to group L on POD21.

### Inhibition of altered Nav1.3, Nav1.7 and Nav1.8 expressions by amitriptyline in injured DRG

**Figure 3:**
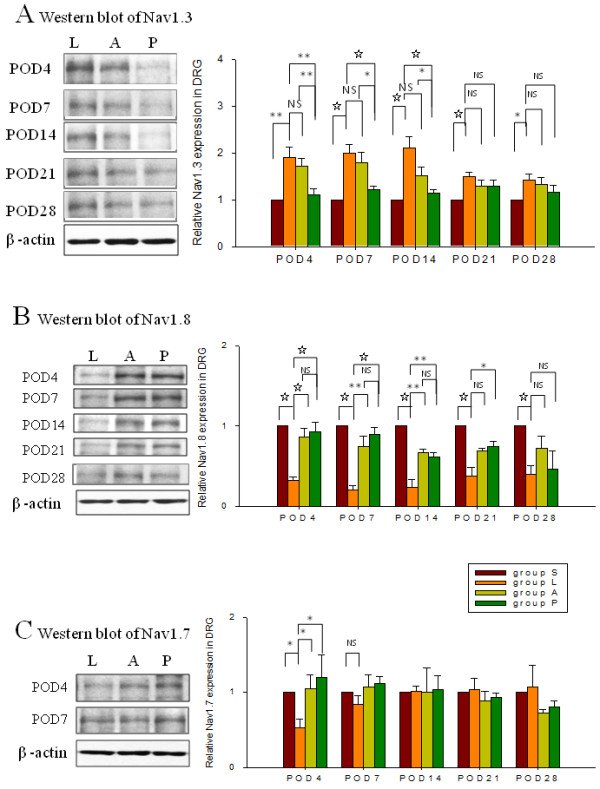
**Protein expressions of Nav1.3, Nav1.8, and Nav1.7 in left L5 DRG by western blot analyses.** Quantification of Nav levels at each time point for each group was normalized against the Nav levels of the sham group. SNL (group L) induced up-regulation of Nav1.3 (A) and down-regulated Nav1.8 (B) for 28 days. Treatment with amitriptyline intra-peritoneally (group A) did not have a significant effect on SNL-induced up-regulation of Nav1.3. Pretreatment with intrathecal amitriptyline together with post-injury treatment with the same compound intra-peritoneally (group P) decreased SNL-induced up-regulation of Nav1.3 for 2 weeks (**A**). Significant inhibition of SNL-induced down-regulated Nav1.8 was found in groups A and P; the effect lasting for 2 and 3 weeks, respectively (**B**). Furthermore, both amitriptyline regimens reversed the SNL-induced down-regulation of Nav1.7 on POD4 (**C**)_._ Data represent mean ± SE. One way ANOVA (n = 24/group, 4-5/each time point). *p < 0.05; **p < 0.01; ☆:*p* < 0.001,NS: not significant. POD: postoperative day; S: Sham; L: Ligation followed by vehicle treatment; A: ligation and treatment with amitriptyline intra-peritoneally (Abdomen) post-injury; P: Pretreatment with intrathecal amitriptyline, ligation and intra-peritoneal amitriptyline post-injury.

Spinal nerve ligation significantly up-regulated Nav1.3 and down-regulated Nav1.8 protein expression for 28 days in injured DRG (Figure 3A and 3B, group S vs. group L). Expression of Nav1.7 in injured DRG was initially down-regulated on POD4 (Figure 3C, group S vs. group L, *p* = 0.026), but gradually returned to baseline level. Treatment with amitriptyline intra-peritoneally for 3 days post-injury preserved Nav1.8 expression for 2 weeks (Figure 3B, *P* = 0.005) but did not attenuate Nav1.3 up-regulation (Figure 3A, *P >* 0.05, group A vs. group L). Pretreatment with intrathecal amitriptyline in addition to the post-injury intra-peritoneal administration not only prolonged the effect of post-injury intra-peritoneal amitriptyline on reversal of Nav1.8 down-regulation for 3 weeks (Figure 3B, *P* = 0.024), but also significantly reduced up-regulation of Nav1.3 for 2 weeks (Figure 3A, *P <* 0.001, group P vs. group L). Treatment with both regimens of amitriptyline (groups P and A) preserved Nav1.7 expression on POD4 when compared with group L (Figure 3C, *P =* 0.033).

### Amitriptyline inhibited glial cell activation in the spinal dorsal horn

**Figure 4:**
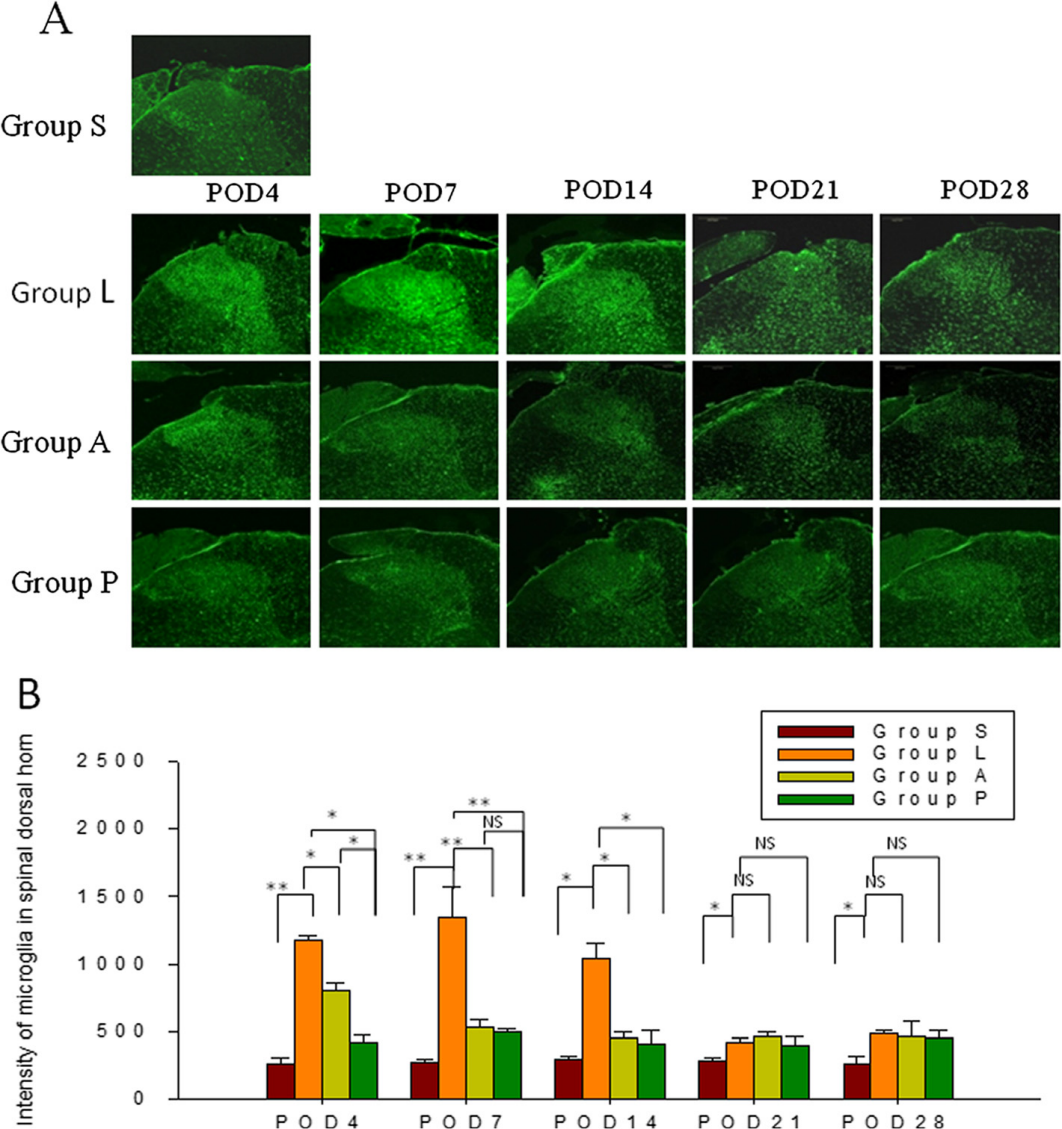
**Immunofluorescence staining (A) and quantification of the expression of OX-42 (B) in microglia of the spinal dorsal horn (SDH).** SNL (group L) induced a dramatic increase in OX-42 positive microglia in the SDH. Both amitriptyline regimens (group A and P) reduced the expression of OX-42 immunoreactivity in microglia for 2 weeks (B). Group P showed a greater effect than group A on reduction of activated microglia in SDH on POD4. Scale bars = 200 μm. Data represent mean ± SE, the Mann–Whitney *U* test was used for statistical comparison. (n = 24/group, 4-5/each time point). *p < 0.05; **p < 0.01, NS: not significant. POD: postoperative day; S: **S**ham; L: **L**igation; A: post-injury intra-peritoneal amitriptyline; P: **P**retreatment with intrathecal amitriptyline in addition to post-injury intra-peritoneal amitriptyline.

**Figure 5:**
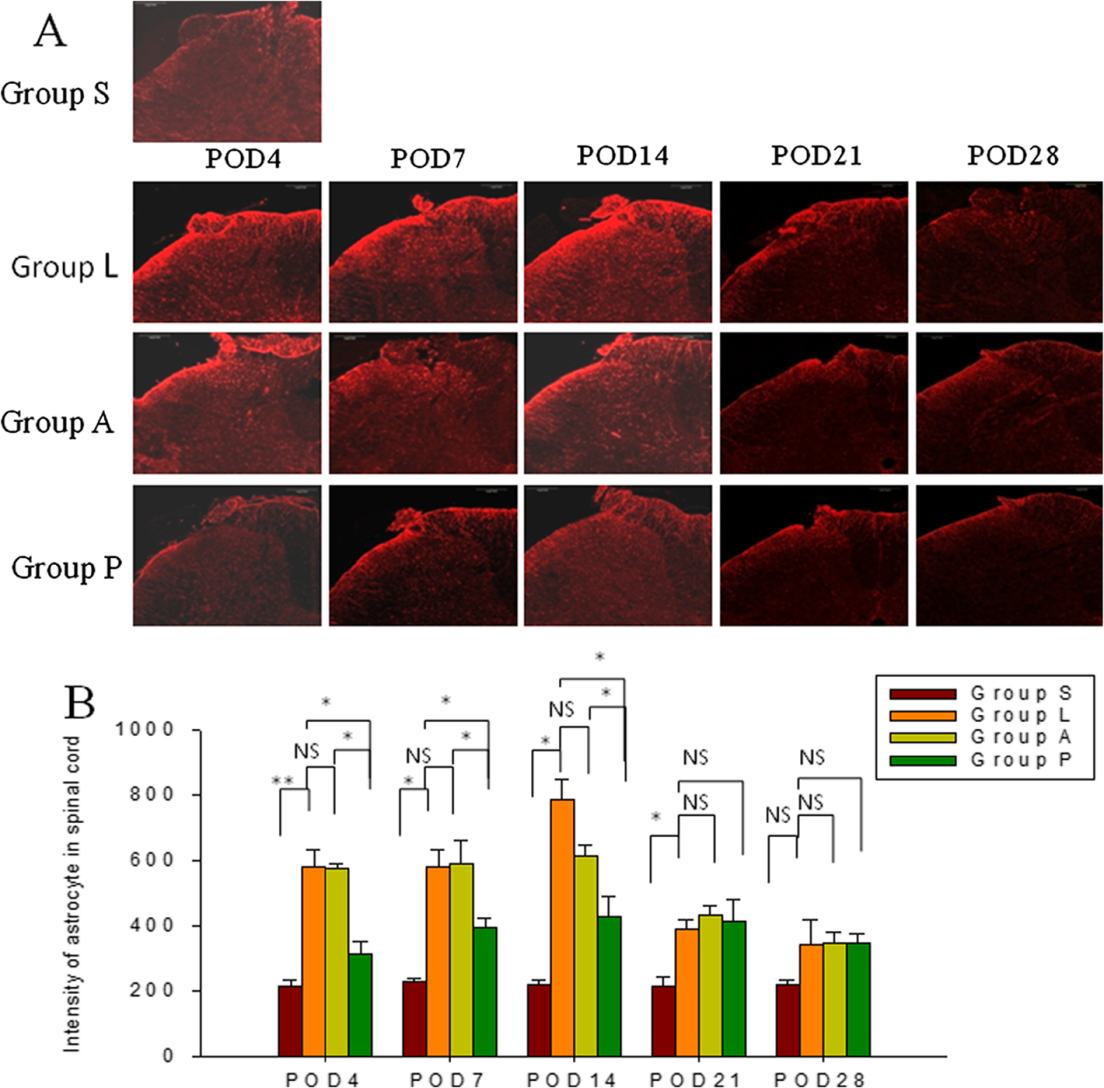
**Immunofluorescence staining (A) and quantification of the expression of GFAP (B) in astrocytes of the spinal dorsal horn (SDH).** Low immunoreactivity was observed in the sham rats (group S). SNL (group L) increased the immunoreactivity of GFAP-positive astrocytes in the SDH. Intra-peritoneal treatment with amitriptyline (group A) had no effect on decreasing the SNL-induced activation of astrocytes. Intrathecal pretreatment in combination with intra-peritoneal administration of amitriptyline (group P) decreased the activation of astrocytes for 2 weeks (group P vs. L). Scale bars = 200 μm. Data represent mean ± SE, the Mann–Whitney *U* test was used for statistical comparison. (n = 24/group, 4-5/each time point). *p < 0.05, ns: not significant. POD: postoperative day; S: **S**ham; L: **L**igation; A: post-injury intra-peritoneal amitriptyline; P: **P**retreatment with intrathecal amitriptyline in addition to post-injury intra-peritoneal amitriptyline.

Spinal nerve ligation resulted in activation of microglia cells and astrocytes in the SDH and the increased activation was most pronounced until POD21 (Figures 4 and 5 group S vs. group L). Administration of amitriptyline intra-peritoneally post-injury reduced the intensity of immunoreactivity induced by SNL in microglia for 2 weeks (Figure 4, *p* = 0.013, group A vs. group L), but had no obvious effect on the activation of astrocytes (Figure 5, group A vs. group L). Addition of intrathecal pretreatment with amitriptyline reduced the increased intensity of microglia activation as well as the activation of astrocytes for 2 weeks (*p <* 0.05).

## Discussion

Peripheral neuropathic pain is characterized by an increased activation of afferent nociceptors and sensitized afferent information through spinal processing [4,24]. Mechanical allodynia, a central sensitization caused by peripheral noxious barrage to the spinal cord, is characterized by a painful sensation evoked by light touch, a normally innocuous stimulation. Touch-evoked pain is a hallmark of neuropathic pain and is triggered by spontaneous ectopic discharges from injured peripheral nerves to sensitized spinal dorsal horn cells. Although mechanisms of allodynia are not entirely understood, they may involve A-beta myelinated afferents [25], activated microglia and astrocytes [26-30], and dorsal horn neuron cells [28]. It has been shown that sodium channel blockers applied to the injured site [31], DRG [32] or the spinal cord [33] all effectively decrease neuropathic pain and attenuate hyperalgesia and allodynia. The present study demonstrates that intrathecal pretreatment with amitriptyline, a sodium channel blocker, enhances the effect of systemic amitriptyline on mechanical and thermal hypersensitivity when compared to systemic administration alone.

Without intrathecal pretreatment, systemic amitriptyline administration showed an effect only on thermal hypersensitivity. The mechanisms by which amitriptyline alleviates hypersensitivity were not determined. Amitriptyline may act, not only as a potent blocker of voltage-gated sodium channels [5] but also as a 5-hydroxytryptamineand noradrenaline reuptake inhibitor, and blocker of α1-adrenergic, nicotinic, muscarinic cholinergic, and N-methyl-D-aspartate receptors [24]. In the present study, the effects of systemic amitriptyline administration to blunt thermal hypersensitivity responses lasted only 7 days. A report by Arsenault and Sawynok [12] showed that a perioperative systemic amitriptyline for 7 days prevents hindpaw hyperalgesic effects for up to 42 days through inhibition of noradrenaline uptake, and increased glial-derived neurotrophic factors as well as brain-derived neurotrophic factors. It also indicates that both pre-injury and post-injury amitriptyline are needed to prevent sensory changes and to blunt chronic neuropathic pain.

Activated glial cells in the dorsal horn have been identified to be closely associated with neuropathic pain [28,34] regardless of whether microglia and astrocytes are activated through cytokines, chemokines, or MAP kinase [34]. Amitriptyline inhibits the secretion of interleukin (IL)-1beta and tumor necrosis factor (TNF)-alpha in rat mixed glial and microglial cell culture [35] and increases expression of anti-inflammatory IL-10 in non-activated microglia [36]. No previous studies have reported attenuation of mechanical and thermal hypersensitivity by amitriptyline associated with a reduction in the amount of activated glial cells in the spinal dorsal horn. We show that post-injury intra-peritoneal treatment with amitriptyline decreases the expression of microglia, while it has no effect on the activation of astrocytes. Interestingly, the effect of systemic amitriptyline was potentiated by intrathecal pretreatment with the compound.

Mechanical allodynia has been demonstrated to be closely associated with activated astrocytes. For example, increased allodynia was found in GFAP-TNF transgenic mice in the L5 SNL animal model [30]. Moreover, dorsal root transection also resulted in intense mechanical allodynia. However, glial responses were almost exclusively in astrocytes, and astrocytic activation was always observed following axotomy and reliably correlated with behavioral responses [29]. Similar to these results, the present study showed that glial cells were activated after nerve damage, and inhibition of glial cell activation was correlated with modulation of mechanical hypersensitivity.

Peripheral nerve axotomy leads to an increased expression in Nav1.3 with a concomitant decrease in Nav1.8 expression in injured DRG neurons [37], and it redistributes sodium channels from sensory neuron soma to peripheral axons at the site of injury [38]. The role of Nav1.3 in neuropathic pain is not clear [21,39,40]. However, an up-regulated Nav1.3 expression in injured DRG is accompanied by the emergence of a rapidly repriming current in these cells and may generate action potentials from a sub-threshold input [41]. On the other hand, the expression of Nav1.8 in injured DRG is significantly decreased, while its expression in adjacent un-injured DRG is increased [37]. In the present study, up-regulated Nav1.3 and down-regulated Nav1.8 were simultaneously found in injured DRG upon peripheral nerve axotomy. These results were significantly reversed after intrathecal pretreatment amitriptyline together with intra-peritoneal post-injury treatment by amitriptyline. These data corroborate our hypothesis that the sodium channel blocker amitriptyline could blunt noxious stimuli transmission, decrease dysregulated expression of voltage-gated sodium channels, and prevent over-excitability and ectopic firing in injured DRG.

Although intrathecal amitriptyline 90 μl of 7.5 mM is tolerable to all rats in our study, and in the study of Chen et al. [19], supra-spinal effects of amitriptyline cannot be excluded from this study. The total volume of rat’s CSF in this study is about 250 μl, calculated from body weight, and intrathecal amitriptyline 90 μl is more than 1/3 of CSF volume. As 90 μl is large volume, the injectate may reach the basal cisterns and ventricles [42]. Further research should be undertaken to elucidate whether the spinal or supra-spinal effects of intrathecal amitriptyline attenuates mechanical hypersensitivity.

Whether intrathecal amitriptyline induces neurotoxicity in the CNS is still unknown. There is no direct neural damage observed for 3% amitriptyline-saline intraspinal administration to dogs, though adhesive arachnoiditis appears [43]. Intrathecal amitriptyline 60 μg in 3 μl was administered via a catheter showing no immediate sensory or motor functional impairment [44]. In rats of morphine tolerance, intrathecal amitriptyline and morphine 15 μg/hr for 5 days maintains anti-nociceptive effect by increase of anti-inflammatory cytokine interleukin-10 expressions [36]. However, high concentrations of amitriptyline infiltrate on the sciatic nerve easily damage peripheral nerve fibers, causing direct injury to axons and producing wallerian degeneration of the nerve fibers [45]. Although intrathecal amitriptyline showed no obvious or extended infiltration of inflammatory cells in spinal cord in the present study, based on evidence in related literature, it is important that low concentrations of amitriptyline be administered either peripherally or centrally to prevent neurotoxicity.

## Conclusion

In the present study, intra-peritoneal administration of amitriptyline twice daily for 3 days following nerve injury attenuated thermal hypersensitivity, reversed down-regulated Nav1.8 and decreased activated microglia, but did not inhibit up-regulation of Nav1.3, mechanical hypersensitivity, or astrocyte activation. However, a combination of intrathecal pretreatment and post-injury intra-peritoneal amitriptyline not only attenuated thermal hypersensitivity but also suppressed mechanical hypersensitivity for one week. It also inhibited the up-regulation of Nav1.3 in injured DRG and reduced the intensity of immunoreactivity in astrocytes induced by SNL. These results suggest that concomitant intrathecal pretreatment and post-injury intra-peritoneal amitriptyline potentiates the attenuation of neuropathic pain than given intra-peritoneally post-injury only.

## Competing interests

The authors declare that they have no competing interests.

## Authors’ contributions

K-I C is the primary author for this manuscript. He conceived, organized, and implemented this research protocol, interpreted the data, guided intellectual discussions of the results, and drafted the manuscript. H-C W and F-Y W are responsible for the integrity of the data and the accuracy of the data analysis. L-L C coordinated the study, interpreted the data, performed the statistical analyses, and drafted the manuscript. C-W C and H-P T executed the study. A-L K and C-S L supervised the research program and contributed to the organized process. All authors have read and approved the final manuscript.

## Financial support

This study was supported by grant from the Kaohsiung Medical University Hospital (KMUH99-9M34).

## Pre-publication history

The pre-publication history for this paper can be accessed here:

http://www.biomedcentral.com/1471-2377/12/44/prepub
